# Achilles’ Heel—The Significance of Maintaining Microenvironmental Homeostasis in the Nucleus Pulposus for Intervertebral Discs

**DOI:** 10.3390/ijms242316592

**Published:** 2023-11-22

**Authors:** Zhangbin Luo, Ziyan Wei, Guangzhi Zhang, Haiwei Chen, Lei Li, Xuewen Kang

**Affiliations:** 1 Department of Orthopedics, Lanzhou University Second Hospital, Lanzhou 730030, China; luozhb21@lzu.edu.cn (Z.L.); 220220904341@lzu.edu.cn (Z.W.); zhanggzh18@lzu.edu.cn (G.Z.); chenhw20@lzu.edu.cn (H.C.); lil21@lzu.edu.cn (L.L.); 2 The Second Clinical Medical College, Lanzhou University, Lanzhou 730030, China; 3 Key Laboratory of Orthopedics Disease of Gansu Province, Lanzhou University Second Hospital, Lanzhou 730030, China

**Keywords:** nucleus pulposus, calcium ion, ASIC, osmotic pressure, metallic elements, intervertebral disc degeneration

## Abstract

The dysregulation of intracellular and extracellular environments as well as the aberrant expression of ion channels on the cell membrane are intricately linked to a diverse array of degenerative disorders, including intervertebral disc degeneration. This condition is a significant contributor to low back pain, which poses a substantial burden on both personal quality of life and societal economics. Changes in the number and function of ion channels can disrupt the water and ion balance both inside and outside cells, thereby impacting the physiological functions of tissues and organs. Therefore, maintaining ion homeostasis and stable expression of ion channels within the cellular microenvironment may prove beneficial in the treatment of disc degeneration. Aquaporin (AQP), calcium ion channels, and acid-sensitive ion channels (ASIC) play crucial roles in regulating water, calcium ions, and hydrogen ions levels. These channels have significant effects on physiological and pathological processes such as cellular aging, inflammatory response, stromal decomposition, endoplasmic reticulum stress, and accumulation of cell metabolites. Additionally, Piezo 1, transient receptor potential vanilloid type 4 (TRPV4), tension response enhancer binding protein (TonEBP), potassium ions, zinc ions, and tungsten all play a role in the process of intervertebral disc degeneration. This review endeavors to elucidate alterations in the microenvironment of the nucleus pulposus during intervertebral disc degeneration (IVDD), with a view to offer novel insights and approaches for exploring therapeutic interventions against disc degeneration.

## 1. Introduction

Low back pain is a prevalent global disease commonly associated with disc degeneration, imposing a significant burden on both society and the economy. With the growing population and aging trend, there has been a rapid increase in the number of individuals experiencing disabilities as a result of lower back pain [1,2]. The intervertebral disc (IVD) is mainly composed of the central nucleus pulposus (NP), the surrounding annulus fibrosus (AF), and the top and bottom cartilage endplate (CEP). IVD is a vital organ in *humans*, which is subjected to external stressors on a daily basis due to the circadian rhythm [3]. Compared to other organs with abundant blood supply, the internal environment of the intervertebral disc (IVD) is relatively enclosed. IVD is a tissue in the *human* body that has fewer blood vessels, and nutrient delivery mainly depends on diffusion through the capillary network in CEP [4]. The CEP is a hyaline cartilage structure that connects the vertebral body to the IVD and comprises hyaline chondrocytes and chondrocytes, which facilitate nutrient delivery to the vascularized IVD [5]. Furthermore, the CEP endures pressure from the IVD in order to safeguard the vertebral body [6]. Due to the difficulty in expelling metabolites and biomechanical limitations, a microenvironment with vascular deficiency, hypoxia, acidity, hypertonicity, and high metabolite accumulation is formed within the nucleus pulposus [6,7,8]. Therefore, in this milieu, the coordination among water molecules, diverse ions, and their respective channels assumes paramount importance. Intervertebral disc degeneration is intricately linked to cellular senescence, inflammatory response, stromal decomposition, endoplasmic reticulum stress, and accumulation of cell metabolites [9,10,11,12,13]. Aquaporin (AQP), calcium ion channels, and acid-sensitive ion channels (ASIC) play a crucial role in regulating the levels of water, calcium, and hydrogen ions that impact various physiological and pathological processes such as cellular aging, inflammatory response, stromal decomposition, endoplasmic reticulum stress, and the accumulation of cellular metabolites. Therefore, it is imperative to investigate the contribution of different ion channels and ions in disc degeneration. Current evidence suggests that Piezo1 channels are implicated in the pathogenesis of intervertebral disc degeneration [14,15,16], while transient receptor potential vanilloid type 4 (TRPV4) also contributes to this process [17,18]. Aquaporin (AQP) and acid-sensitive ion channels (ASIC), as channels for basic physiological functions of cells, also affect intervertebral discs in different ways [7,8,19,20,21,22,23,24,25,26,27,28,29,30,31,32,33,34,35]. Additionally, tension response enhancer binding protein (TonEBP) plays a crucial role in regulating osmotic pressure within the disc [21,36,37,38,39]. Furthermore, calcification of the disc cartilage endplate and the distribution of potassium, zinc, tungsten, and iron within the disc also impact its function [40,41,42,43,44,45,46]. This review serves as a valuable resource for comprehending the alterations in the microenvironment of the nucleus pulposus during disc degeneration and for devising potential therapeutic interventions.

## 2. Regulation of Calcium Ion Homeostasis and Calcium Ion Channels

Intracellular calcium signaling is widely recognized as one of the most extensively investigated second messenger pathways [47]. Calcium ions not only serve as signal transmitters in the cytoplasm, but also play a crucial role in intracellular organelles such as mitochondria, nucleus, and endoplasmic reticulum [12]. Ca^2+^ can be regarded as a versatile messenger that operates at various levels, including cellular, subcellular, and extracellular domains [48]. As a second messenger, Ca^2+^ plays a crucial role in transmitting signals for various fundamental physiological processes such as cell cycle regulation, survival, programmed cell death, migration, and gene expression [49]. The maintenance of intracellular Ca^2+^ homeostasis plays a pivotal role in regulating critical cellular processes such as survival, growth, differentiation, metabolism, and apoptosis [50]. Several *human* diseases are associated with dysregulation of Ca^2+^ homeostasis, including developmental disorders [51], hypertension [52], cardiovascular disease [53], diabetes [54], Alzheimer’s disease [55] and cancer [56,57]. The endoplasmic reticulum (ER) and mitochondria serve as the primary intracellular Ca^2+^ storage organelles, releasing Ca^2+^ through either the 1,4,5-triphosphate receptor (IP3R) or the ryanodine receptor (RyR) in response to various stimuli that generate intracellular Ca^2+^ signaling. This signaling pathway is responsible for regulating nearly every aspect of cellular function [58,59].

The concentration of Ca^2+^ is precisely regulated by a variety of processing enzymes, proteins, channels, and transporters that work together to fine-tune the time- and spatially-specific Ca^2+^ signal [60]. Inositol 1,4,5-triphosphate (IP3) acts as a second messenger primarily produced through the metabolism of inositol 4,5-diphosphate (PIP2) by phospholipase C (PLC). Upon binding to IP3R, it facilitates the rapid release of Ca^2+^ that has accumulated in the endoplasmic reticulum and other cell membranes [61]. IP3R facilitates the transport of Ca^2+^ across the endoplasmic reticulum membrane and induces Ca^2+^ release from both endoplasmic reticulum and mitochondria, thereby elevating cytoplasmic Ca^2+^ concentration [62]. The RyR is a distinctive intracellular Ca^2+^ release pathway located in the endoplasmic reticulum of cells, which regulates the flow of Ca^2+^ from intracellular reservoirs such as the endoplasmic reticulum into the cytoplasm. Activation of RyR occurs through an intracellular increased Ca^2+^-induced Ca^2+^ release (CICR) mechanism [63]. The sarcoplasmic/endoplasmic reticulum Ca^2+^ ATPase (SERCA) actively reuptakes released Ca^2+^ back into the sarcoplasmic/endoplasmic reticulum to maintain proper Ca^2+^ homeostasis [60]. The IP3R, RyR, and SERCA proteins play crucial roles in calcium transfer between the endoplasmic reticulum and cytoplasm. Specifically, IP3R and RyR facilitate the release of Ca^2+^ from the ER into the cytoplasm, while SERCA is responsible for actively transporting Ca^2+^ back into the ER from the cytoplasm. Together, they ensure a coordinated balance of Ca^2+^ outflow from and inflow into the endoplasmic reticulum [64,65].

The calcium ion, being a crucial regulator of cellular function, also plays a pivotal role in the process of intervertebral disc degeneration. Studies have demonstrated that the concentration of Ca^2+^ in CEP tissues increases concomitantly with the progression of intervertebral disc degeneration. The vertebrae in direct contact with CEP are likely to be the primary source of elevated Ca^2+^ levels within CEP. As the medium’s Ca^2+^ concentration rises, there is a corresponding concentration-dependent decrease in the expression of collagen I, collagen II, and aggrecan in CEP cells, along with an increase in the activity of catabolic enzyme ADAMTS-5. Additionally, in the case of an increase in Ca^2+^, there will be a decrease in the diffusion of glucose into CEP and NP tissues, as well as a reduction in the expression of proteoglycan in CEP tissues, leading to CEP calcification. However, calcium-induced disc degeneration can be reversed through co-culturing with cells using calcium-sensitive receptor (CaSR) antagonists or by suppressing the expression of CaSR [41]. The study conducted by another research study highlighted a negative correlation between serum calcium concentration and the extent of disc degeneration, indicating that lower levels of serum calcium are associated with more severe disc degeneration [66]. Regardless of the veracity, these two studies demonstrate the involvement of changes in Ca^2+^ concentration in disc degeneration.

The Ca^2+^ not only functions extracellularly, but also serves as a crucial intracellular second messenger. The studies have demonstrated that within the articular cartilage tissue and IVD, when the pH value falls within the physiological range, Ca^2+^ exhibits a remarkably strong affinity towards ASICs and can bind to the lateral side of the ASICs channel pore, consequently inducing channel closure. Upon pH reduction, H+ attach themselves to acid-sensitive sites on ASICs, thereby diminishing Ca^2+^’s binding affinity towards ASICs. As a result, Ca^2+^ dissociates from the channel pore, facilitating ASIC opening and subsequent cellular entry [67]. When exposed to a pH 6.0 medium, which mimics the acidic microenvironment of degenerative discs, there was a significant increase in intracellular Ca^2+^ flow in bone marrow mesenchymal stem cells (BMSCs). Lower pH levels activated ASIC1a, leading to the infiltration of Ca^2+^ into BMSCs. An acid-induced increase in Ca^2+^ influx leads to Ca^2+^-mediated activation of calpsin and calcineurin. The cytosolic phosphatase calcineurin, activated by calpsin, plays a crucial role in cellular apoptosis. Activation of calpsin by Ca^2+^ further stimulates calcineurin, thereby facilitating caspase-mediated apoptosis [26]. Previous studies have indicated that Ca^2+^ plays a role in acid-induced damage to NPCs through ASIC1a channels. The expression of ASIC1a was significantly upregulated in degraded NPCs compared to healthy ones. When NPCs were cultured under acidic conditions, there was a notable increase in intracellular Ca^2+^, which could be reduced by the presence of psalmotoxin-1 (PcTX1), an ASIC1a blocker, or the absence of extracellular Ca^2+^. ASIC1a may potentially contribute to intervertebral disc degeneration. Activation of ASIC1a leads to an elevation in intracellular Ca^2+^ flux in NPCs, thereby playing a crucial role in NPCs necrosis, apoptosis, and stress-induced premature aging during the process of intervertebral disc degeneration [7]. In addition, culture in an acidic environment (pH = 6.2) suppressed the proliferation of nucleus pulposus mesenchymal stem cells (NP-MSCs), increased the rate of apoptosis, and upregulated gene expression of ASICs subunits (*ASIC1*, *ASIC2*, *ASIC3*, and *ASIC4*). The expression levels of ECM-related genes (*aggrecan*, *collagen II*, and *SOX-9*) were downregulated, while the expression levels of stem cell-related genes (*Oct4*, *Nanog*, *Jagged*, and *Notch1*) were also decreased. Sa12b is a *wasp* peptide that inhibits Ca^2+^ influx to counteract the effects caused by acidic conditions [32]. In addition to an acidic environment, the concentration of Ca^2+^ in NPCs also significantly increased following hypoxia treatment. Hypoxia treatment induces alterations in Ca^2+^ concentration and upregulates ASIC3 expression. Furthermore, hypoxia and ASIC3 overexpression exert inhibitory effects on NPC proliferation, arresting the cell cycle at the G1 phase, promoting apoptosis, initiating autophagy, and upregulating HIF-1α and LC3 expression [8]. The findings suggest that Ca^2+^ plays a crucial role in regulating the microenvironment of NPCs and influencing their growth under hypoxic conditions.

The involvement of Ca^2+^ in endoplasmic reticulum stress induced by mechanical load has been highlighted in several studies. After prolonged mechanical loading, the endoplasmic reticulum stress (ERS) pathway is activated, leading to the binding of endoplasmic reticulum and mitochondria. At the interface between these two organelles, an IP3R-GRP75-VDAC1 complex exists. This complex facilitates a significant upregulation of Ca^2+^ levels in NPCs, from the endoplasmic reticulum to mitochondria. The elevated Ca^2+^ levels then polarize mitochondria, resulting in a reduction of intracellular ATP levels. Additionally, this process leads to the release of lactate dehydrogenase (LDH) and high mobility group protein 1 (HMGB1) into extracellular mediators. The process of exchanging Ca^2+^ between the endoplasmic reticulum and mitochondria leads to cellular bioenergy depletion and triggers compression-induced programmed necrosis in NPCs [12]. DNA repair and protein-modifying enzymes (PARP) play crucial roles in regulating various cellular activities, including cell repair, DNA transcription and replication, cytoskeletal organization, and protein degradation, among others [68]. Apoptosis-inducing factor (AIF), which is localized in mitochondria and released in response to death stimuli, plays a crucial role in mediating apoptosis [69]. During the compression of NPCs, Ca^2+^ mediates the activation of PARP and induces an increase in cleaved PARP (C-PARP) expression. Additionally, Ca^2+^ facilitates the translocation of AIF from mitochondria to cytoplasm and, subsequently, into the nucleus. By promoting the PARP-AIF pathway, Ca^2+^ plays a crucial role in mediating programmed necrosis of NPCs under stress conditions. Additionally, compression can induce reactive oxygen species (ROS) accumulation in NP cells, leading to enhanced endoplasmic reticulum stress and mitochondrial Ca^2+^ overload that ultimately triggers programmed cell necrosis [12]. In another study, the upregulation of G protein-coupled receptor 35 (GPCR35) was observed in response to mechanical stress or ROS, leading to a significant increase in intracellular influx of Ca^2+^ and upregulated levels of intracellular ROS. These series of changes contribute to the down-regulation of collagen II and aggrecan expression, as well as an exacerbation of IVD degeneration [70].

In addition to mechanical loading, advanced glycation end products (AGEs) also impact calcium homeostasis. AGEs are proteins or lipids that undergo glycation after exposure to sugar and are present in various cell types, affecting both extracellular and intracellular structure and function [71]. Treatment with AGEs can induce ERS, leading to a significant increase in cytoplasmic Ca^2+^ concentration and a significant decrease in ER lumen Ca^2+^ concentration, as well as activation of downstream apoptotic pathways and an increased rate of cellular apoptosis [72]. Studies have demonstrated a significant increase in cytoplasmic Ca^2+^ levels, IP3R, RyR, and IP3 expression in severely degraded NP cells. Conversely, there is a notable decrease in endoplasmic reticulum Ca^2+^ levels and SERCA activity. In terms of regulating ER Ca^2+^ release, inhibiting IP3R proves more effective than inhibiting RyR activity to prevent subcellular calcium redistribution and subsequent ER stress-induced apoptosis. They hypothesized that inhibiting the IP3R-mediated release of calcium from the endoplasmic reticulum could further suppress the RyR-induced release of calcium (known as Ca^2+^-induced Ca^2+^ release or CICR) [65,73]. Treatment with AGEs disrupts the regulation of intracellular calcium channels and pumps in the endoplasmic reticulum, leading to imbalances in intracellular calcium levels and subsequent activation of ER stress and apoptosis pathways. Blocking the release of calcium from the endoplasmic reticulum can improve the subcellular redistribution of calcium, alleviate ER stress, and reduce apoptosis [65]. In conclusion, maintaining homeostasis of endoplasmic reticulum Ca^2+^ can serve as a protective mechanism against AGEs-induced IVD degeneration, while inhibiting the release of endoplasmic reticulum Ca^2+^ may ameliorate AGEs-induced IVD degeneration.

Transient receptor potential vanilloid type 4 (TRPV4) is a non-selective cation channel that responds to osmotic pressure and mechanical stimulation, allowing for the permeation of Ca^2+^, Na^+,^ and Mg^2+^ ions [74]. The activation of TRPV4, which is crucial for regulating intracellular Ca^2+^ concentration and maintaining intracellular water balance, can be induced by mechanical stimulation, low osmotic pressure, as well as exposure to hot and acidic environments [75]. Studies have demonstrated the expression of TRPV4 in intervertebral discs, with immunohistochemistry revealing its presence in both nucleus pulposus and annulus fibrosus cells of *bovine* tail IVDs. In *humans*, degraded IVDs exhibit increased TRPV4 expression within the NP region compared to healthy discs, which is further promoted by decreased osmotic pressure. This heightened TRPV4 expression leads to an increase in intracellular calcium flux and interleukin-1β (IL-1β) production in IVD cells [18]. In addition, the TRPV4 channel serves as a crucial mechanosensor in AF cells. Treatment of AF cells with the TRPV4 agonist GSK101 resulted in an elevation of intracellular calcium levels and enhanced stress fiber formation in AF cells. When the cells were pre-treated with the TRPV4 antagonist GSK219, the calcium response induced by GSK101 was inhibited in AF cells. Moreover, both cyclic tensile strain (CTS) and TRPV4 activation with GSK101 were observed to enhance the expression of aggrecan and proteoglycan 4 in AF cells. Furthermore, the overexpression of aggrecan and proteoglycan 4 induced by CTS was found to be inhibited by GSK219. These findings suggest that TRPV4 serves as a crucial sensor for detecting CTS in AF cells [17]. After being subjected to external mechanical stimulation, TRPV4 functions as a receptor, facilitating an increase in intracellular calcium flux. This process converts physical signals into chemical signals, with Ca^2+^ serving as the second messenger within the cell for further transmission of information. Notably, TRPV4 plays a pivotal role in regulating both calcium flux and matrix metabolism in AF cells.

Additionally, Ca^2+^ plays a crucial role in disc degeneration through its involvement in the Piezo1 channel. The study revealed that Piezo1 is an ion channel with seven transmembrane domains [76]. The *FAM38A* gene encodes Piezo1, a newly discovered mechanosensitive cation channel that facilitates the non-selective permeation of divalent ions such as Ca^2+^, Mg^2+^, and Mn^2+^ [77]. When the nucleus pulposus cells are mechanically stretched, the Piezo1 channel is upregulated, facilitating an increase in Ca^2+^ influx and thereby converting mechanical signals into chemical signals. This rise in intracellular calcium concentration triggers activation of the NF-κB pathway, leading to enhanced expression of NLRP3 and pro IL-1β. The Piezo1 inhibitor GsMTx4 attenuates the mechanically-induced upregulation of NLRP3 components, including NLRP3, ASC, caspase-1, and IL-1β, in nucleus pulposus cells [14]. Ca^2+^ serves as a mediator of the inflammatory response triggered by Piezo1 activation through mechanical stretching, ultimately leading to intervertebral disc degeneration. In addition to mechanical stretching, extracellular matrix stiffness can also act as a mechanical stimulus that activates Piezo1 channel opening and regulates Ca^2+^ influx. The latest research findings suggest that the activation of Piezo1 is triggered by extracellular matrix stiffness, resulting in an increase in intracellular calcium levels and ROS, which subsequently induces endoplasmic reticulum stress and ultimately leads to senescence and apoptosis of nucleus pulposus cells [15]. A team of researchers has developed a cell stimulation device, utilizing a glass micropipette (micropipette ultrasound), which delivers ultrasonic waves through the tip of the pipette to elevate intracellular calcium ion levels in nucleus pulposus cells. The team demonstrated the involvement of mechanosensitive GPCRs and associated calcium signaling pathways in NP cells’ response to micropipeted ultrasound, with both ASIC3 channel and mechanosensitive GPCRs contributing to the increase in intracellular calcium. However, inhibition of the Piezo channel did not show any effect on intracellular calcium signaling [78]. The differential intracellular calcium signal response following Piezo channel inhibition may be attributed to the distinct effects of mechanical stretch and ultrasonic stimulation on cells, as well as the varying responses of Piezo channels to different stimuli.

**Figure 1:**
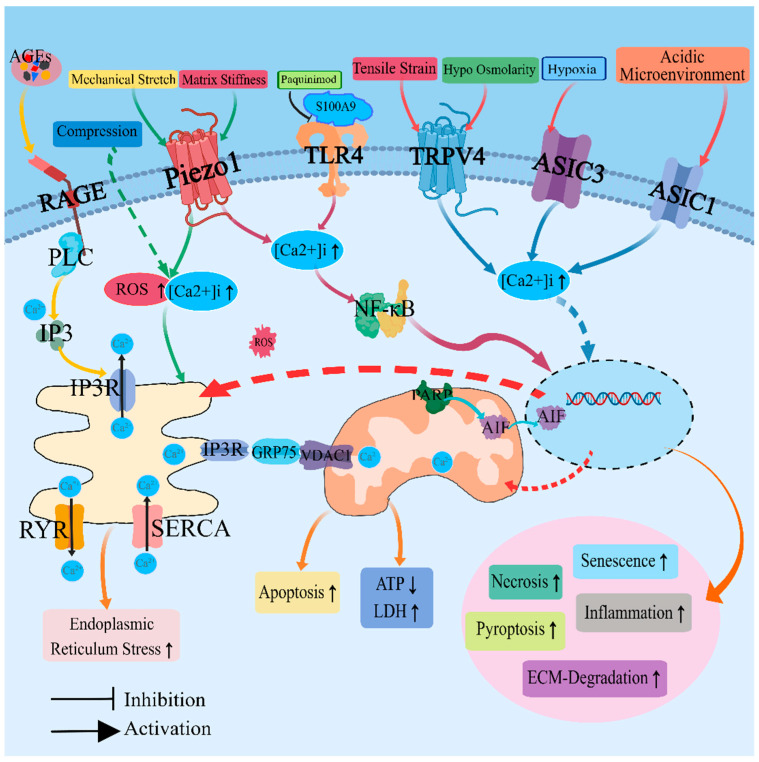
The action of various factors on nucleus pulposus cells induces changes in calcium ion levels and triggers a cascade of subsequent regulatory processes. PLC: phospholipase C; IP3: inositol 1,4,5-triphosphate; IP3R: 1,4,5-triphosphate receptor; RYR: ryanodine receptor; SERCA: sarcoplasmic/endoplasmic reticulum Ca^2+^ ATPase; ROS: reactive oxygen species; PARP: DNA repair and protein-modifying enzymes; AIF: apoptosis-inducing factor; ECM: extracellular matrix.

The S100 protein family, a group of small acidic calcium-binding proteins, functions as either calcium sensors or extracellular factors to regulate diverse biological processes [79]. It has been implicated in tumor metastasis [80], cell proliferation [81], various acute and chronic inflammatory conditions [82], oxidative stress [83], and apoptosis [84]. S100A9 functions as a calcium sensor, undergoing conformational changes upon binding to calcium ions and thereby regulating calcium-dependent signaling pathways. Its expression is strongly induced during various inflammatory processes such as trauma, infection, heat stress, and others, with inflammation being a major source of its secretion [85]. The expression of calcium-binding protein S100A9 was significantly increased in the degenerative nucleus pulposus tissues. Calcium-binding protein S100A8/A9 interacts with Toll-like receptor 4 (TLR4) to activate the NF-κB signaling pathway, resulting in a significant upregulation of matrix-degrading enzymes MMP-3 and ADAMTS-4, a significant down-regulation of matrix proteins aggrecan and collagen II, and an increase in apoptosis of NPCs. It also enhances the expression of inflammatory cytokines IL-1, IL-6, IL-8, and TNF-α, leading to IVDD. Moreover, inhibitors targeting S100A8/A9 prevent IVDD development and inflammation-related pain in *rat* models [86,87] (Figure 1).

Connective tissue growth factor (CTGF/CCN2) is a multifunctional protein belonging to the CCN family of stromal cell proteins, which governs cellular processes including proliferation, differentiation, adhesion, and various other biological phenomena. It actively participates in disease-associated pathways such as the Hippo pathway, p53 signaling, and NF-κB pathways, thus acting as a downstream effector that contributes to the development of inflammation, fibrosis, cancer progression, and other pathological conditions [88,89,90,91]. The expression of CCN2 protein is regulated by Ca^2+^. In NPCs, ionomycin treatment increases intracellular Ca^2+^ concentration, leading to the activation of downstream calcineurin (CaN) and nuclear factor T (NFATs/TonEBP). Subsequently, CaN and NFATs promote CCN2 activity and expression. Thus, the extracellular signal is transmitted intracellularly through the Ca^2+^-CaN/NFAT-CCN2 signaling pathway to enhance CCN2 protein expression [38].

In addition to influencing intervertebral disc degeneration, dysregulation of Ca^2+^ concentration also contributes to the sensitization of dorsal root ganglion (DRG) neurons in a hypoxic and acidic environment. Hypoxia and acidosis can enhance the spontaneous calcium response in *sheep* DRG neurons. The direct exposure of DRGs to hypoxia and low pH resulted in a more pronounced increase in calcium flux compared to the indirect, simulated disc stimulation of DRGs. Transient elevations in cytoplasmic calcium concentrations reflect neuronal firing activity, while the proportion of cells exhibiting spontaneous calcium responses indicates the possibility of “spontaneous firing”. Notably, the growth spurt is more susceptible to alterations in calcium signaling than the neuronal cell body [92]. The transient elevation of intracellular calcium levels following neuronal depolarization constitutes a crucial step in the exocytosis of neurotransmitters, which are indispensable for the synaptic transmission of noxious or nociceptive signals to the central nervous system [93]. The aforementioned studies have substantiated the pivotal role of Ca^2+^ in nerve signal transduction. In summary, Ca^2+^ exerts a significant influence on regulating cellular physiological functions and forms a complex regulatory network. Precise regulation of Ca^2+^ is crucial for maintaining the stability of the microenvironment of nucleus pulposus cells (Table 1).

## 3. Revisions in the Acidic Environment and Acid-Sensitive Ion Channels

Maintaining acid-base balance is crucial for normal cellular physiology, yet various pathological conditions including ischemia, hypoxia, inflammation, and cancer can disrupt pH homeostasis [35]. The H^+^ ion is a crucial component of the cellular environment, and alterations in its concentration can significantly impact cellular physiological functions. Acid-sensitive ion channels (ASICs) and H^+^ play pivotal roles in regulating these functions. At least seven subtypes of ASIC in mammals—ASIC1a, ASIC1b, ASIC2a, ASIC2b, ASIC3, ASIC4, and ASIC5—are reported to be encoded by five genes (*Accn1*, *Accn2*, *Accn3*, *Accn4*, and *Accn5*) [27]. Each subtype of ASIC consists of two transmembrane domains with intracellular N- and C-terminals, as well as a large extracellular ring. The crystallization of the *chicken* ASIC1a channel reveals that a functional channel necessitates the presence of three subunits. The composition of these subunits within the functional channel can either be homomorphic, consisting of three identical ASIC subtypes, or heteromorphic, comprising different combinations of ASIC subtypes with distinct electrophysiological properties [28]. ASICs are primarily cation channels gated by protons, which can be activated by a decrease in pH [31]. Numerous studies have indicated that ASICs are also implicated in the transmission of mechanical signals [27,28,30,31,94]. The ASICs are capable of mediating various nociceptive and chemosensory signal transmission, including skin injury, muscle injury, heart ischemia and angina, visceral injury, bone injury, as well as acidosis, decreased oxygen levels, Ca^2+^ levels alteration, and high osmotic pressure [30]. The activation of ASICs has been implicated in a diverse range of pathological conditions, encompassing ischemic brain injury, substance dependence, neurogenic inflammation, chronic pain syndromes, hypertension, heart failure, gastroesophageal reflux disease, irritable bowel syndrome, interstitial cystitis, hearing loss, tooth sensitivity, and imbalance of body balance [27,31].

The intervertebral disc is the largest avascular tissue in the body [95]. Research has demonstrated that the extracellular environment pH in healthy IVDs is maintained between 7.1 and 7.4, while severely degraded discs typically exhibit a reduced pH of 6.5, and surgically excised diseased IVD tissues can have a pH as low as 5.7 [96,97,98]. The intervertebral disc microenvironment is characterized by the attraction of a multitude of cations, including H^+^ ions, due to the presence of negatively charged proteoglycans, leading to an acidic milieu. Furthermore, degenerated discs exhibit elevated lactate production as a consequence of pro-inflammatory cytokine activity [7]. Additionally, the intervertebral disc receives nutrients through the cartilage endplate, while glycolysis serves as the sole provider of cellular energy. However, inadequate nutrient diffusion and excessive accumulation of lactic acid can lead to an acidic microenvironment within the IVD [95,99,100]. Lactic acid is a byproduct of anaerobic glycolysis in cells, and its accumulation within the intervertebral disc creates an acidic microenvironment that plays a crucial role in regulating cellular behavior. Specifically, lactic acid has the ability to modulate the matrix composition of NPCs and may also act as a key regulator of autophagy and apoptosis during NPC degeneration. The findings of previous studies have demonstrated that the presence of a high-concentration lactic acid culture environment can induce autophagy in NPCs, leading to a significant increase in the number of TUNEL-positive cells. Additionally, this environment significantly upregulates the expression levels of activated caspase-3 and caspase-3, while concurrently downregulating sulfated glycosaminoglycan (sGAG) and collagen II expression levels. Consequently, there is a notable decrease in extracellular matrix synthesis [100]. Moreover, ASIC1a and ASIC3 are pivotal in the progression of intervertebral disc degeneration triggered by a lactic acid milieu. After exposure to lactic acid, stimulation of NPCs by lactic acid leads to upregulation of ASIC1a and ASIC3 expression levels, resulting in calcium influx, promotion of intercellular ROS levels, subsequent activation of the NF-κB signaling pathway, ECM degradation promotion, and induction of the inflammatory component NLRP3. This cascade also induces an increase in caspase-1 expression, IL-1β production, LDH level elevation, and GSDMD-N release, ultimately leading to pyroptotic cell death [34]. In the distinctive milieu of the intervertebral disc, the progressive accumulation of lactic acid gradually exacerbates the degenerative progression of the intervertebral disc.

ASICs play a crucial role in modulating the microenvironment of nucleus pulposus cells and influencing their growth. Within the acidic milieu of the intervertebral disc, ASICs contribute to some extent to its degeneration. The expression levels of ASIC1 and ASIC3 gradually increase in degraded NP, while acidic conditions also induce upregulation of ASIC1 and ASIC3 expression in NP [25,101]. Moreover, the hypoxic microenvironment can induce alterations in intracellular Ca^2+^ levels within NPCs, leading to the upregulation of ASIC3 expression and activation of the MAPK pathway. Consequently, this cascade results in cell cycle arrest at the G1 phase, inhibition of NPCs proliferation, initiation of apoptosis and autophagy processes, as well as upregulation of HIF-1α and LC3 expressions [8]. The expression of ASIC has been consistently demonstrated in numerous studies to exhibit a positive correlation with the catabolism of nucleus pulposus cells. Moreover, acidic conditions significantly induce an upregulation of ASIC3 expression, thereby leading to augmented matrix degradation in NPCs. This acid-induced response is characterized by a substantial reduction in aggrecan expression, an elevation in *MMP-3* and *ADAMTS-4* gene expression, as well as a notable decrease in *TIMP-2* expression [29]. Similarly, comparable outcomes were observed in endplate chondrocytes, whereby an acidic environment activates ASIC1a and subsequently triggers the NF-κB signaling pathway to impact the stromal metabolism of endplate chondrocytes, leading to a reduction in collagen II and aggrecan expression while upregulating the expression of stromal catabolic enzymes MMP-1, MMP-9, and MMP-13 [33]. The acidic environment also upregulated the levels of MMP-3 and MMP-9 in nucleus pulposus mesenchymal stem cells (NP-MSCs), while suppressing the expression of collagen I, collagen II, aggrecan, and SOX-9 associated with extracellular matrix synthesis. This led to an enhanced breakdown of the extracellular matrix [101,102]. However, treatment with ASIC inhibitors (amiloride, PcTx1, or APETx2) effectively reversed these effects and partially alleviated the catabolism of the nucleus pulposus matrix [29,101]. Similarly, using ASIC1a-siRNA or PDTC (pyrrolidine-dithiocarbamate, an inhibitor of NF-κB) resulted in a reversal of matrix degradation induced by acidic conditions. Interestingly, inhibition of ASIC1a-siRNA did not prevent acid-induced upregulation of MMP-3 expression [33].

**Figure 2:**
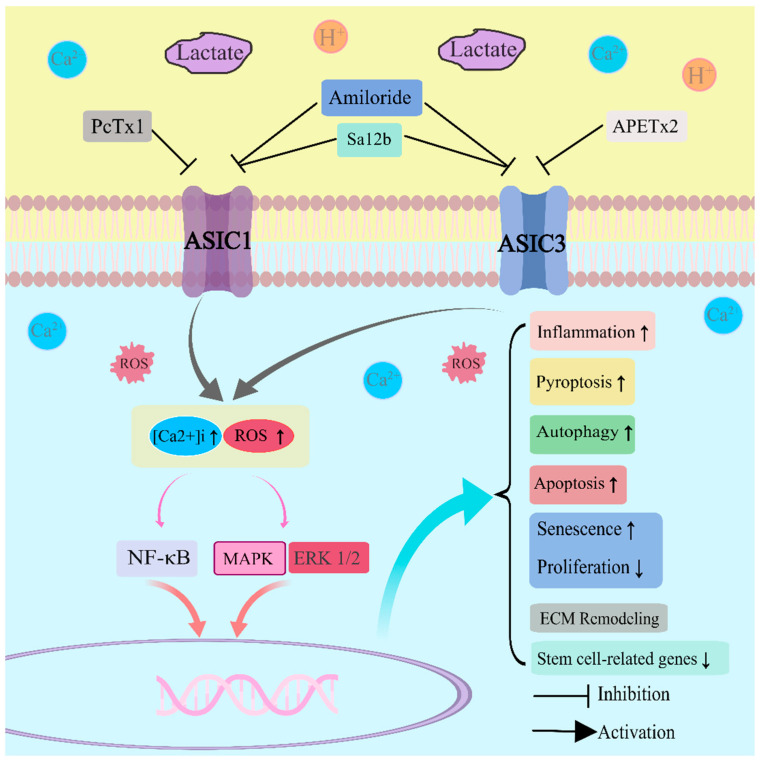
Distinct mechanisms of action for ASIC inhibitors and the implications of ASICs activation. ROS: reactive oxygen species; NF-κB: nuclear factor kappa-B; MAPK: mitogen-activated protein kinase; ERK: extracellular regulated protein kinases; ECM: extracellular matrix.

Furthermore, an acidic environment and upregulation of ASIC expression can induce inflammation and senescence in nucleus pulposus cells. The acidic microenvironment hinders the cell cycle progression of NP cells, leading to arrest in the G0/G1 phase. Consequently, there is a significant increase in the proportion of cells in the G1 phase along with elevated expression levels of p53, p21, and p16 proteins associated with aging. Additionally, IL-6 and IL-8 levels are also elevated, ultimately accelerating cellular aging [7,25]. Moreover, an acidic environment induces increased expression of ASIC1 and ASIC3 in NP-MSCs while suppressing stem cell-related genes (*Oct4*, *Nanog*, *Jag1*, and *Notch1*) [101]. However, inhibition of ASIC3 expression using APETx2 reduces the expression of inflammatory cytokines such as IL-1β and IL-6 as well as pain-related factors like nerve growth factor (NGF) and brain-derived neurotrophic factor (BDNF) [29]. After blocking ASIC1a with PcTX1, the degree of cell senescence was also reduced [7]. Similarly, the utilization of the ASICs inhibitor amiloride significantly augmented the cellular proliferation rate, alleviated the suppression of stem cell-associated genes under acidic conditions, and preserved the differentiation potential of NP-MSCs [101]. The acidic environment is a detrimental factor that inhibits the bioactivity of *human* NP-MSCs during intervertebral disc degeneration. Amiloride has been shown to protect NP-MSCs from acid-induced damage by blocking ASICs. Apoptosis also plays an important role in intervertebral disc degeneration [103,104]. Acidic culture conditions lead to a significant increase in Ca^2+^ levels, ASIC expression, LDH release, and cell damage in NPCs. Additionally, it stimulates apoptosis of NPCs by increasing the expression of Bax and cleaved caspase-3 proteins while decreasing the expression of Bcl-2. However, upon blocking the ASICs, the aforementioned process is reversed [7,8,101]. Similarly, acidic culture activates ASIC1a channels in cartilage endplate cells leading to increased intracellular Ca^2+^ concentration and eventual apoptosis which can be halted by blocking ASIC1a channels [105] (Figure 2).

Sa12b, a *wasp* peptide, has been shown to effectively inhibit ASIC currents in *rat* dorsal root ganglion neurons [106]. Additionally, Sa12b can suppress the expression of AISCs in *human* nucleus pulposus mesenchymal stem cells (hNP-MSCs), decrease intracellular Ca^2+^ influx, and protect hNP-MSCs from proliferation inhibition and increased apoptosis caused by an acidic environment culture [32]. Some studies have linked Sa12b to the C-terminal of RADA16-I and constructed a RAD/SA1 biomaterial containing Sa12b, which can enhance the expression of aggrecan, collagen II, and SOX-9 in hNP-MSCs under acidic conditions. Meanwhile, it inhibits the expression of P-ERK protein and reduces intracellular Ca^2+^ levels to suppress apoptosis [107]. In *sheep* dorsal root ganglion neurons, the combination of hypoxia and low pH led to a significantly enhanced spontaneous calcium response during neurite growth and augmented calcium response to bradykinin in DRG neurons compared to normoxic conditions and neutral pH [92]. The acidic environment of the intervertebral disc or increased ASIC expression can induce detrimental effects on nerve roots, rendering DRG sensitive to fluctuations in calcium signaling, heightening pain sensitivity, and promoting mechanical hyperalgesia. These factors may contribute to the development of low back pain in patients with intervertebral disc herniation.

ASICs are susceptible to harmful factors such as an acidic microenvironment and lactic acid, which trigger subsequent processes that promote cellular adaptation to the harsh conditions of degenerated discs. In summary, an acidic environment can exacerbate intervertebral disc degeneration by increasing necrosis of NPCs, impairing proliferation leading to cell senescence, enhancing apoptosis, and augmenting extracellular matrix catabolism. Throughout this degenerative process, ASICs act as accomplices in disc degeneration caused by an acidic environment. Blocking ASIC receptors or inhibiting their expression could potentially delay the progression of disc degeneration. Acidic culture conditions can decrease the activity of NPCs, enhance cell matrix catabolism, and initiate cellular inflammation. Therefore, inhibiting ASICs may represent a promising approach for treating IVDD (Table 2).

## 4. Alterations in Osmotic Pressure and Associated Regulatory Proteins

Aquaporins (AQPs) play diverse roles in mammals, and their involvement in the transport of water and solutes is crucial for the proper functioning of various organ systems and cell types [108,109]. The expression of AQPs is also detected in nucleus pulposus cells to some extent, yet it gradually declines with the aging of intervertebral discs [22,23,24]. Immunohistochemical staining of the intervertebral disc in *rats* revealed a gradual decrease in AQP-1 and AQP-3 expression with the progression of IVDD. In 2-month-old *rats*, AQP-1 and AQP-3 were predominantly localized in the inner layer of NP and AF, while weak immunostaining for AQP-1 and AQP-3 was observed in the outer region of AF. However, in 18-month-old *rats*, there was a significant reduction in the number of cells positive for both AQP-1 and AQP-3 within the IVD [22]. Studies have demonstrated a gradual decrease in AQP-1 expression within adult and elderly *rabbit* lumbar NPs, with extracellular osmotic pressure and oxygen concentration exerting an influence on its regulation. Specifically, decreased osmotic pressure of the cellular environment resulted in down-regulation of AQP-1 expression, while decreased oxygen concentration led to upregulation. Notably, the expressions of collagen II and aggrecan were significantly reduced under conditions of decreased osmotic pressure or oxygen concentration; moreover, the combination of both factors led to a more pronounced reduction [23]. AQP-1 exhibits oxygen permeability and the upregulation of AQP-1 under hypoxic conditions facilitates enhanced oxygen diffusion into cells to mitigate hypoxic damage [19]. Therefore, it is reasonable to induce upregulation of AQP-1 in NP cells exposed to hypoxia. In addition to relying on glycolysis for energy production, NPCs can also respond to an anoxic environment by upregulating the expression of AQP-1 [23]. The findings on *human* nucleus pulposus cells suggest a potential association between intervertebral disc degeneration and reduced expression of AQP-3. Inhibition of AQP-3 expression resulted in suppressed hNPCs proliferation and intensified extracellular matrix catabolism, while overexpression of AQP-3 inhibited the Wnt/β-catenin signaling pathway, significantly increased hNPCs proliferation, promoted aggrecan expression, and markedly decreased the expression of matrix-degrading enzymes ADAMTS-4 and ADAMTS-5 [24].

TonEBP, also known as activated T-cells nuclear Factor 5 (NFAT5), is implicated in the pathogenesis of various diseases including rheumatoid arthritis, atherosclerosis, diabetic nephropathy, acute kidney injury, hyperlipidemia and insulin resistance, autoimmune diseases such as type 1 diabetes and multiple sclerosis, salt-sensitive hypertension, and hepatocellular carcinoma [110,111,112,113,114]. TonEBP plays a crucial role in the regulation of extracellular osmotic pressure and the maintenance of stromal homeostasis in intervertebral discs and articular cartilage [36]. The presence of TNF-α facilitates the nuclear translocation of TonEBP protein; however, TNF-α-mediated TonEBP activation does not elicit the upregulation of osmotic pressure regulatory genes. Furthermore, the inflammatory milieu associated with intervertebral disc degeneration does not impede TonEBP’s ability to regulate osmotic pressure [37]. Inflammation is recognized as a contributing factor to disc degeneration [115]. However, disc degeneration can also be induced by osmotic pressure through distinct mechanisms independent of inflammation. The investigation revealed that the expression levels of TonEBP were abundant in NPCs of 12-month-old *mice* but decreased in 22-month-old *mice*. Moreover, TonEBP-deficient *mice* exhibited fibrocartilage alterations and elevated levels of MMP-13, which are characteristic features of disc degeneration. Notably, TonEBP deficiency synergistically impairs disc health with aging [39].

Studies have demonstrated that exposure to a hypertonic environment significantly upregulates the expression of AQP-1 and AQP-5 in hNPCs, as well as AQP-1 in *rat* NPCs. However, the knockdown of TonEBP expression reversed the hypertonicity-induced expression of both AQPs. Furthermore, fluorescence staining for AQP-1 and AQP-5 in the NP region was markedly reduced in TonEBP knockout *mice* [21]. TonEBP regulates the expression of AQP-1 and AQP-5 in intervertebral discs under hypertonic conditions. The expression of connective tissue growth factor (CTGF/CCN2) in nucleus pulposus cells is regulated not only by calcium ions, but also by TonEBP. In NP cells, with the increase in osmotic pressure in the environment, the expression level of TonEBP increased, but the expression level of CCN2 decreased. Further studies have confirmed that inhibiting TonEBP activity can rescue the inhibition of CCN2 in a hypertonic microenvironment, and that TonEBP mediates the regulation of CCN2 under such conditions [38]. It has been demonstrated that exposure to a low osmotic pressure environment upregulates the expression of TRPV4, IL-1β, and IL-6 in NPCs. Inhibition of TRPV4 expression attenuated the hypotonic-induced increase in gene expression of *IL-1β* and *IL-6*, indicating that TRPV4 signaling contributes to pro-inflammatory cytokine production in NPCs [18]. Aggrecan is the primary proteoglycan in intervertebral discs and plays a crucial role in determining their osmotic pressure [116]. The proteoglycan content in degenerated intervertebral discs is comparatively lower than that in healthy IVDs, and exposure to pro-inflammatory cytokines induces the degradation of proteoglycans, leading to reduced osmotic pressure within the IVDs. The low osmotic pressure environment facilitates the upregulation of TRPV4 ion channels, leading to an augmented secretion of pro-inflammatory cytokines and thereby establishing a detrimental cycle of intervertebral disc degeneration [18].

Notochordal cells (NCs) are the remnant cells of the notochords of all chordates originating in early embryogenesis and located in the center of the intervertebral disc [117,118]. The immature nucleus pulposus consists of cells of notochordal origin that are larger and contain an extensive cytoskeletal network and many vacuoles, which are lost with age and are thought to be important in regulating metabolic changes that may lead to age-related disc degeneration [119]. It has been pointed out that vacuolar notochordal cells are very sensitive to microenvironmental stimulation, and static hyperosmolar overload can lead to the differentiation of NCs into small NP cells (sNPCs) and promote glycosaminoglycan accumulation. During the maturation of NC, due to changes in osmotic pressure and mechanical pressure, cell-to-cell N-cadherin (N-cad) decreases and connexin 43 (Cx-43) protein increases, and NPCs can adapt to osmotic pressure changes by changing osmotic regulatory protein, mechanotransduction protein, and glycosaminoglycan synthesis [20]. The intervertebral disc is subjected to pressure from internal and external environments every day, and maintaining a complete disc shape is a strong guarantee to reduce disc degeneration. As a relatively closed structure, it is extremely important to keep the osmotic pressure inside the nucleus pulposus relatively stable. Moderate osmotic pressure will promote the maturation of nucleus pulposus cells and the accumulation of extracellular matrix, while excessive osmotic pressure will affect the expression of osmotic pressure correlators such as AQPs, TonEBP, and CCN2, causing aseptic inflammation of the intervertebral disc microenvironment, leading to the degradation of the extracellular matrix, and eventually the degeneration of the intervertebral disc. Therefore, it is extremely important to maintain the relative stability of the osmotic pressure in the nucleus pulposus microenvironment.

## 5. Repartition and Deposition of Metallic Elements

Although the intervertebral disc is a relatively enclosed environment, its components exhibit complexity. *Human* disc specimens consistently demonstrated the highest concentrations of iron, zinc, and copper, irrespective of degeneration status. Moreover, notable disparities in copper, magnesium, potassium, and calcium were observed between degraded discs and healthy ones [120]. The concentration of glycosaminoglycan, including chondroitin sulfate (CS) and hyaluronate (HA), is found to be higher in the nucleus pulposus compared to the intervertebral disc margin (annulus fibrosus) according to several studies. Additionally, there is a greater presence of Na^+^ and K^+^ ions in the central region of the disc with lower concentrations at its boundaries. However, despite this disparity, the nucleus pulposus in the center of the disc contains fewer cells than the annulus fibrosus. The expression levels of HA and CS exhibit a positive correlation with sodium and potassium ion concentrations while cell density shows a negative correlation with them [43]. These findings suggest that metal elements and ions may potentially regulate the physiological functions of nucleus pulposus cells.

The two-pore domain potassium channel TREK-1 is predominantly expressed in the central nervous system (CNS) of *humans*. It exhibits high expression levels in the brain, spinal cord, heart, kidney, ovary, and small intestine and is implicated in various physiological and pathological processes such as neuroprotection, epilepsy, depression, and pain perception [121]. Evidence suggests that TREK-1 is also present in the nucleus pulposus and annulus fibrosus cells of *human* intervertebral discs [40]. Although the specific role of TREK-1 channels in intervertebral discs remains unclear, they may contribute to disc degeneration as mechanically activated ion channels that can be stimulated by an acidic environment.

Additionally, several studies have suggested that Zn^2+^ also plays a role in the degeneration of intervertebral discs. In nucleus pulposus cells, IL-1β can enhance the influx of Zn^2+^ into the cells, which subsequently leads to an increase in matrix metalloproteinases, indicating that elevated Zn^2+^ influx may contribute to the progression of IVDD [44]. Recent studies have shown that drinking water containing low doses of sodium tungstate for four weeks significantly reduced lumbar disc height in *mice*, accompanied by decreased glycosaminoglycan content and increased fibrosis. Tungsten also increases the expression of inflammatory cytokines IL-1β and TNF-α, as well as NGF and BDNF in intervertebral discs, while increasing pain marker calcitonin gene-related peptide (CGRP) expression [42], suggesting its potential involvement in IVDD progression. Studies have demonstrated that the GSK-3β inhibitor LiCl can activate the Wnt/β-catenin signaling pathway by stabilizing β-catenin, leading to apoptosis of nucleus pulposus cells and degradation of extracellular matrix [122]. However, pretreatment with an appropriate concentration of LiCl in a strategy for treating disc degeneration by transplanting adipose-derived stem cells (ADSCs) resulted in increased proliferative capacity and NP-labeled expression. Additionally, LiCl pretreatment induced an increase in ROS levels and ERK1/2 activation, which reduced cell death and promoted extracellular matrix deposition [123]. The findings of this experiment are remarkable and indicate the advantageous role of metallic elements in the management of disc degeneration.

In a study of 217 patients with low back pain, it was discovered that serum ferritin levels can serve as an indicator for the severity of IVDD, with those having serum ferritin levels below 170.5 ng/mL exhibiting more severe IVDD [124]. The same survey revealed a significant decrease in iron levels in the blood of individuals with IVDD compared to healthy subjects, and this reduction in iron concentration was found to be progressively associated with the severity of IVDD. Furthermore, analysis of degraded nucleus pulposus specimens demonstrated a significant down-regulation of the iron-containing gene *PolE*, as well as the catalytic subunit of DNA polymerase epsilon (*Polε*), along with two other *Polε* subunits, namely *PolE2* and *PolE3*. Conversely, certain proteins involved in apoptosis, such as caspase-3 and caspase-8, exhibited a notable upregulation [125]. In addition to the reduction of iron in blood, excessive accumulation of iron in NPCs is also a significant contributor to intervertebral disc degeneration. Immunohistochemical analysis of degraded *human* nucleus pulposus specimens revealed decreased expression of the iron transporter (FPN). Moreover, the down-regulation of FPN results in iron overload in *human* NPC cells, which is involved in TBHP-induced cell death and IVDD development [126]. Other studies have demonstrated that iron overload can induce the expression of stromal degrading enzymes in endplate chondrocytes and decrease the expression of collagen II in a dose-dependent manner. Oxidative stress, mediated by iron overload, can promote osteogenic differentiation and mineralization of endplate chondrocytes. Furthermore, iron overload also contributes to mitochondrial dysfunction and ferroptosis in endplate chondrocytes. Iron overload is closely associated with the pathogenesis of intervertebral disc degeneration through oxidative stress and ferroptosis [46]. Therefore, mitigating oxidative stress or inhibiting ferroptosis may represent promising therapeutic strategies for the management of intervertebral disc degeneration.

## 6. Remodeling and Calcification of the Cartilage Endplate

**Figure 3:**
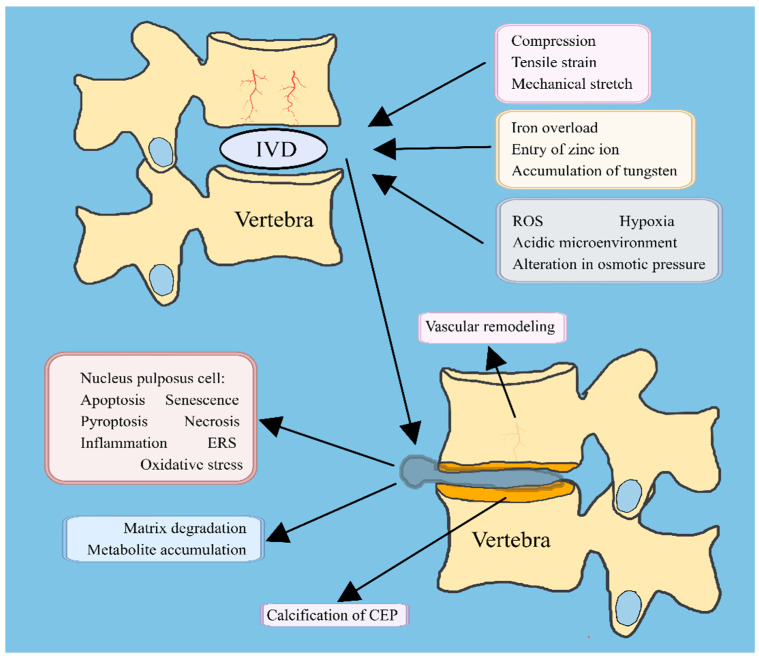
Changes in the microenvironment of the nucleus pulposus resulted in alterations to both the nucleus pulposus cells and the surrounding intervertebral disc structure. IVD: intervertebral disc; CEP: cartilage endplate; ERS: endoplasmic reticulum stress.

The hardening of the cartilage endplate and decreased permeability are considered significant factors contributing to disc degeneration [127]. In a *rabbit* model of disc degeneration induced by fine needle puncture, it was observed that the cartilage endplate underwent remodeling, the vasculature changed with disc degeneration, and there was reduced transport of small molecules into the degenerative disc [128]. Calcium-sensitive receptors (CaSRs) are a subclass of G-protein-coupled receptors (GPCRs) that play crucial roles in calcium signal transduction, maintenance of calcium homeostasis, and parathyroid hormone secretion [129]. The expression of CaSR was significantly upregulated in degraded cartilage endplate cells. In the presence of elevated levels of extracellular calcium, CaSR can mediate the upregulation of alkaline phosphatase (ALP), which is a major enzyme involved in tissue mineralization in CEP. Additionally, the synthesis of matrix proteins collagen I, collagen II, and aggrecan is decreased by CaSR activation, and this partially mediates the degeneration of CEP [41]. Treatment with H_2_O_2_ in cartilage endplate cells enhances the activity of alkaline phosphatase (ALP) and promotes the expression of RUNt-related transcription factor 2 (RUNX2), osteocalcin (OCN), and collagen I, which are involved in the maturation and vascularization of cartilage. Furthermore, H_2_O_2_ treatment reduces levels of inorganic pyrophosphate (PPi) while increasing levels of inorganic phosphate (Pi). PPi effectively inhibits calcium/phosphate crystal nucleation and propagation, whereas Pi promotes mineralization [45]. These findings suggest that oxidative stress may exert regulatory control over calcification processes and enhance chondrocyte mineralization by augmenting the Pi content. In addition, iron overload is an independent risk factor for disc degeneration. Iron overload can cause oxidative stress in CEP cells, promote the expression of endplate chondrocyte stroma-degrading enzymes, decrease the expression of collagen II in a dose-dependent manner, accelerate the degeneration of endplate chondrocytes, and eventually lead to degeneration and mineralization of endplate chondrocytes [46]. In conclusion, the cartilage endplate serves as the sole conduit for nutrient supply to the nucleus pulposus. Remodeling of the intervertebral disc’s cartilage endplate can alter both the nutritional status and microenvironment of the nucleus pulposus, while calcification of this structure may accelerate intervertebral disc degeneration (Figure 3).

## 7. Future Directions and Conclusions

In this article, we present a summary of the changes in the microenvironment of the nucleus pulposus during intervertebral disc degeneration. The microenvironmental homeostasis of the nucleus pulposus plays a crucial role in protecting the disc, as the disc is subjected to daily internal and external pressures. Environmental and internal pressures can affect various factors such as aquaporin (AQP), calcium and acid-sensitive ion channels (ASIC), Piezo1, transient receptor potential vanilloid type 4 (TRPV4), tension response enhancer binding protein (TonEBP), and metallic elements that influence the microenvironment where the nucleus pulposus resides. Various environmental factors, such as increased levels of reactive oxygen species or hypoxia, elevated calcium concentrations, accumulation of cellular metabolites, altered osmotic pressure, and the presence of metallic elements, are more or less present in the microenvironment surrounding the nucleus pulposus. Alterations in the microenvironment of the nucleus pulposus can induce cellular aging, apoptosis, inflammation, extracellular matrix degradation, endoplasmic reticulum stress, ferroptosis, and other pathological changes within nucleus pulposus cells. Furthermore, this may lead to cartilage endplate calcification and exacerbate the already compromised microenvironment of the nucleus pulposus, thereby establishing a vicious cycle. Disc degeneration, an age-related disease, has become increasingly prevalent. While there are numerous treatments available for this condition, the key to successful treatment lies in reducing adverse factors within the disc environment while maintaining a normal internal environment.

The management of age-related disc degeneration involves various strategies aimed at reducing or mitigating the progression of disc degeneration. Hypertension, inadequate physical activity, and gout have been identified as potential factors contributing to disc degeneration, according to a study [130]. Furthermore, there exists a correlation between obesity and the degeneration of intervertebral discs [131]. The presence of diabetes is widely recognized as a significant risk factor that demonstrates a causal association with IVDD [132,133]. Smoking is also implicated asogenesis of intervertebral disc degeneration [134,135,136]. Therefore, engaging in regular physical activity, refraining from smoking, avoiding obesity, and maintaining optimal metabolic function may constitute crucial strategies for preserving intervertebral disc health. Currently, there exists a plethora of treatments available for disc degeneration. Platelet-rich plasma (PRP) has emerged as an efficacious modality in the management of disc degeneration [137,138]. Presently, PRP has been extensively employed in the therapeutic intervention targeting disc degeneration. In one clinical study, patients with discogenic low back pain who were injected with platelet-rich plasma within the intervertebral disc showed significant improvement in the intensity of their low back pain and improved walking ability, although no change was shown on the nucleus pulposus MRI [139]. Numerous fundamental studies have consistently indicated that mesenchymal stem cells represent a promising therapeutic for the management of disc degeneration [140,141,142]. Autologous adipose mesenchymal stem cells (AT-MSC) combined with hyaluronic acid were implanted into the intervertebral disc for the treatment of patients suffering from chronic discogenic low back pain. At the 1-year follow-up post surgery, patients who received this combination therapy demonstrated significant improvements in Visual Analogue Scale (VAS), Oswestry Disability Index (ODI), and Short Form-36 (SF-36) scores. Additionally, three patients exhibited an increase in water content within the nucleus pulposus based on diffuse MRI findings [143]. Furthermore, exosomes derived from mesenchymal stem cells have demonstrated the potential to mitigate H_2_O_2_-induced intervertebral disc degeneration, apoptosis of nucleus pulposus cells, and degradation of extracellular matrix [144]. Simultaneously, these exosomes derived from mesenchymal stem cells exhibit an inhibitory effect on pyroptosis by targeting the NLRP3 pathway [145]. Furthermore, in a prospective cross-sectional study, it was observed that 11% of the 290 patients who underwent disc removal surgery due to disc disease were found to be infected with Propionibacterium acnes [146]. The bacterium Propionibacterium acnes has been demonstrated to induce apoptosis of NPCs and trigger mitochondria-mediated cell death via the TLR2/c-Jun N-terminal kinase (JNK) pathway, while also activating autophagy and facilitating extracellular matrix degradation [147]. Furthermore, Propionibacterium acnes can induce inflammation and cell death in NPCs via the NLRP3 inflammasome-mediated pyroptosis pathway [148]. Therefore, the inhibition of Propionibacterium acnes could potentially offer therapeutic benefits in the management of intervertebral disc degeneration. Moreover, traditional Chinese medicines have been demonstrated to play a prominent role in maintaining homeostasis of the inner nucleus pulposus and inhibiting disc degeneration [149,150,151,152]. Additionally, owing to their resemblance to natural extracellular matrix and exceptional biocompatibility, hydrogels exhibit immense potential as controlled drug release vectors for the treatment of disc degeneration. Various drug-loaded hydrogels present promising therapeutic prospects for disc degenerative diseases [153,154,155,156]. Regrettably, numerous treatments remain in the experimental stage and necessitate further investigation prior to their implementation in clinical settings. Although we possess a fundamental comprehension of the correlation between alterations in the nucleus pulposus microenvironment and intervertebral disc degeneration, numerous inquiries remain unanswered regarding the mechanism of modifications in the nucleus pulposus microenvironment during the onset and progression of intervertebral disc degeneration.

## Figures and Tables

**Table 1:** Alterations in calcium ions and associated regulatory proteins.

Cellular Phenotype	Stimulating Factor	Protein Involved in the Process	Roles of Elevated Ca^2+^	Measures to Mitigate Effects Caused by Ca^2+^ Elevation	References
*Rat*-NPCS	Ionomycin	—	[Ca^2+^]_i_↑ CCN2↑	BAPTA-AM FK506 and CsA	[38]
*Bovine*-NPCS	Micro-pipette ultrasound	ASIC3 GPCRs	[Ca^2+^]_i_↑	Amiloride APETx2 GPAnt-2 U73122	[78]
HNPCS	Compression loading Zaprinst H_2_O_2_	GPR35↑	[Ca^2+^]_i_↑	GRP35-siRNA	[70]
*Bovine*-NPCS	GSK101 Hypo-osmotic environment	TRPV4↑	[Ca^2+^]_i_↑	—	[18]
*Mice*-annulus fibrosus cells	GSK101	TRPV4↑	[Ca^2+^]_i_↑	GSK2193874 Y-27632	[17]
HNPCS	Matrix stiffness	Piezo1↑	[Ca^2+^]_i_↑ ERS↑ Apoptosis↑ Senescence↑	Piezo1-siRNA	[15]
HNPCS	Mechanical stretch	Piezo1↑	[Ca^2+^]_i_↑ NLRP3, ASC, caspase-1, and IL-1β↑ NF-κB↑	BAPTA-AM	[14]
*Rat*-BMSC	Culture media with pH 6.0	ASIC1a↑ calpain↑	Proliferation↓ Mitochondrial apoptosis↑	PcTX1 calpeptin cyclosporine-A	[26]
HNP-MSCS	Culture media with pH 6.2	ASIC1 ASIC2 ASIC3 ASIC4	[Ca^2+^]_i_↑	Sa12b Amiloride	[32]
*Human*-synovial fibroblasts	Culture media with pH 6.0	ASIC1a↑ NFATc3↑	RANTES, IL-8, MIP-1a, ICAM-1, sTNF-RI, and sTNF-RII↑	ASIC1a-shRNA PcTx-1 extracellular calcium chelator- EGTA	[67]
HNPCS	AGEs	IP3R↑ RyR↑ SERCA↓	[Ca^2+^]_i_↑ [Ca^2+^]_er_↓ ERS↑ Apoptosis↑	U73122 Xec Rya	[65]
*Rat*-NPCS	Excessive compression loading	IP3R–GRP75–VDAC1 Complex↑	[Ca^2+^]_m_↑ ERS↑ HMGB1↑ LDH↑ ATP↓ C-PARP ↑ Nu-AIF↑	4-PBA Xestospongin-C VDAC1-siRNAGRP75-siRNA Ruthenium red (RR) NAC	[12]

[Ca^2+^]_i_: intracellular Ca^2+^; [Ca^2+^]_er_: endoplasmic reticulum luminal Ca^2+^; [Ca^2+^]_m_: mitochondrial Ca^2+^; C-PARP: cleaved DNA repair and protein-modifying enzymes; Nu-AIF: apoptosis-inducing factor of the nucleus. The upward arrows(↑) indicate that the expression of the protein or gene is increased or up-regulated; The downward arrows(↓) indicate that the expression of the protein or gene is reduced or down-regulated.

**Table 2:** The roles of ASICs in intervertebral disc degeneration.

Cellular Phenotype	Stimulating Factor	Quantity of ASICs	ASICs Inhibition	Roles of Blocking ASICs	References
HNPCS	Culture media with different pH levels	ASIC1a↑	PcTx1	LDH↓ Apoptosis↓ Senescence↓	[7]
HNPCS	Culture media with different pH levels	ASIC3↑	APETx2	IL -1β, IL -6, NGF, and BDNF↓	[29]
HNPCS	Different concentrations of lactate	ASIC1a↑ ASIC3↑	Amiloride PcTx1 APETx2	ASIC1, ASIC3↓ NLRP3, caspase-1, and IL-1β↓ Pyroptosis↓ ROS, LDH↓	[34]
HNP-MSCS	Culture media with pH 6.6	ASIC1↑ ASIC3↑	Amiloride PcTx1 APETx2	ASIC1, ASIC3↓ Proliferation↑ Recovery of the cell cycle↑ IL-6, IL-8↓ p53, p21, p27, rb1, and p16↓ Senescence↓ Collagen II, Aggrecan↑ MMP-3, MMP-9↓	[25]
HNP-MSCS	Culture media with different pH levels	ASIC1↑ ASIC2↑ ASIC3↑ ASIC4↑	Amiloride	Proliferation↑ Apoptosis↓ Inhibited the expression of ASICs Notch1, Jagged, Nanog, and Oct4↑ Collagen I, Collagen II, Aggrecan, and SOX-9↑	[101]
HNP-MSCS	Culture media with pH 6.2	—	Sa12b RAD-Sa12b hydrogels Amiloride	Proliferation↑ Apoptosis↓ ASIC1, ASIC2, ASIC3, and ASIC4↓ Collagen II, Aggrecan, and SOX-9↑ Oct1, Nanog, Jagged, and Notch12↑ Influx of Ca^2+^↓ Collagen I↓ P-ERK↓	[32,107]
*Rabbit*-NPCS	2% oxygen Overexpression of ASIC3	ASIC3↑	shRNA-ASIC3	ASIC3↓ Proliferation↑ Apoptosis↓ Autophagy↓ LC3↓ HIF-1α↓ ERK1/2 and MAPK↓	[8]
*Rat*-endplate chondrocytes	Culture media with pH 6.0	ASIC1a↑	ASIC1a-siRNA PcTx1	Collagen II, Aggrecan↑ MMP-1, MMP-9, and MMP-13↓ NF-κB↓ Apoptosis↓ Influx of Ca^2+^↓	[33,105]

LDH: lactate dehydrogenase; NGF: nerve growth factor; BDNF: brain-derived neurotrophic factor; ROS: reactive oxygen species; MMP: matrix metalloproteinase. The upward arrows(↑) indicate that the expression of the protein or gene is increased or up-regulated; The downward arrows(↓) indicate that the expression of the protein or gene is reduced or down-regulated.
